# Rheological Properties of Styrene-Butadiene-Styrene Asphalt Mastic Containing High Elastic Polymer and Snow Melting Salt

**DOI:** 10.3390/polym14173651

**Published:** 2022-09-02

**Authors:** Yangsen Cao, Jiarong Li, Zhuangzhuang Liu, Xinzhou Li, Fan Zhang, Baozeng Shan

**Affiliations:** 1 School of Highway, Chang’an University, Xi’an 710064, China; 2 Key Laboratory for Special Area Highway Engineering of Ministry of Education, Chang’an University, South 2nd Ring Road Middle Section, Xi’an 710064, China

**Keywords:** road engineering, salt storage pavement, salt storage asphalt mastic, high elastic polymer, rheological properties

## Abstract

Sprinkled snow melting salt (SMS) exerts a snow melting effect and also has a negative impact on the asphalt pavement and the environment. Salt storage pavement technology can alleviate these two problems. However, non-alkaline SMSs may have the risk of affecting asphalt mastic properties and further affecting the mechanical properties of asphalt pavements. Therefore, the general properties and rheological properties of two styrene-butadiene-styrene-modified asphalts with and without high elastic polymer were studied after adding SMS. The asphalt mastic without a high elastic agent is defined as the SBS group, and the other group is the HEA group. Our results show that the HEA group shows a lower penetration and a higher softening point, ductility, and viscosity than the SBS group. The more the SMS, the more the reduction effect of the general performance. The elastic recovery of asphalt mastic decreases with the content of SMS. SMS has no obvious effect on the ratio of the viscous and elastic composition of asphalt mastic. The creep of asphalt mastic increases with the content of SMS. The high elastic polymer can significantly reduce the creep, and even the strain of HEA100 is smaller than that of SBS00. SMS increases the creep stiffness and reduces the creep rate at low temperature. Although SMS increases the potential of asphalt pavement to melt ice and snow, it also reduces the high-temperature rutting resistance and low-temperature crack resistance of asphalt mastic. Salt storage pavement materials can be used in combination with high elastic polymers to reduce the negative effects brought by SMSs.

## 1. Introduction

Economic development is inseparable from the support of transportation. As one of the two most widely used types of pavements, asphalt pavement has the characteristics of driving comfort and low noise. In winter, however, snowfall or standing water can easily lead to icy roads. The icing on the road reduces the friction between the pavement and the tires, further causing brake failure. The icing of the asphalt road brings a huge safety hazard to traffic. Ensuring the safety and smoothness of traffic in ice and snow weather has always attracted the attention of road practitioners.

To keep traffic safe in winter, researchers have tried a variety of strategies to clear snow and ice from roads. In general, these strategies can be roughly divided into two types, one is the external snow removal method, and the other is the internal snow removal method [1]. The external ice and snow removal method adopts the traditional ice and snow removal technology, which mainly involves manual ice and snow removal, mechanical ice and snow removal, and spreading snow melting agents. Internal ice and snow removal methods mainly include the thermal snow melting method [2], the elastic deicing method [3], the suppress freeze method [4], and the conductive concrete method [5]. These three methods of the traditional ice and snow melting methods are generally used in synergy to improve the efficiency of ice and snow removal. However, the large-scale distribution of SMS is labor-intensive, especially in less mechanized areas. In addition, the amount of SMS is difficult to control, which leads to the waste of SMS on the one hand, and the surge of maintenance costs on the other hand [6]. Regardless of the cost, the high-concentration salt solution formed by the ice and snow melting water combined with the SMS will not only have an irreversible impact on pavement materials but also harm the soil, causing soil salinization and endangering the ecological environment. Furthermore, is that this top-down ice-melting strategy has difficulty solving the freezing between the pavement and the ice. The distribution of SMS needs to be combined with the weather forecast; otherwise, traffic jams will still occur even if snow removal machinery is used [7]. To reduce the impact of traditional ice and snow melting technology on the pavement and the environment, researchers have gradually turned their attention to the more promising internal ice and snow removal method, that is, active ice and snow removal technology [8].

The thermal ice melting and conductive concrete technology in the active ice and snow removal technology belong to the energy conversion ice and snow melting technology. Thermal self-melting technology is widely used in heating cable ice melting technology [9] and heat pipe ice melting technology [10]. The conductive concrete technology mainly adds conductive substances such as graphite [11], carbon fiber [12], and steel fiber [13] to the pavement material, and then increases the temperature of the pavement through resistance heat dissipation to melt ice and snow. These two types of energy-converting ice and snow melting technologies have remarkable effects, but they have not been widely used due to the disadvantages of the required large investment and high energy consumption. The elastic de-icing and snow pavement is to add elastic material to the mixture to break the icing of the pavement through the elastic deformation of the pavement. Elastic de-icing is theoretically feasible, but the effect is limited in practical application [14]. The freeze-inhibiting pavement to remove ice and snow is to achieve the purpose of removing ice and snow by inhibiting the formation of ice on the pavement surface. Specific practices include the use of superhydrophobic surface coatings to reduce ice adhesion and accumulation [15], and the use of rough surface structures to inhibit ice formation [16]. The method of inhibiting the formation of ice layers reduces the possibility of pavement surface freezing from the root, but it has the characteristics of high cost and poor wear resistance.

The traditional method of de-icing and snow removal is effective, but there are problems such as untimely de-icing, environmental pollution, and large labor consumption. In active ice and snow removal technology, the energy conversion type of ice and snow melting technology has high energy consumption and high cost. Resilient pavement de-icing increases pavement flexibility and may increase vehicle fuel consumption. The pavement technology to inhibit freezing is still immature and insufficiently popularized. In view of the various problems existing in the above-mentioned ice and snow melting technology, salt storage pavement ice and snow removal technology came into being [17,18,19]. This technology adds slow-release salt storage materials to pavement materials in the form of fillers. The formation of ice is delayed or inhibited for a period of time by the gradual migration of salts to the pavement surface through the compression of vehicle tires, vibration, and the action of water. Salt storage pavement has great potential in the field of ice and snow melting due to its timely removal of ice and snow, controlled amount of SMS, reasonable construction cost, and the same construction method as traditional pavement [20].

In the research on salt storage pavement, Tan [21] studied the performance of a salt storage asphalt mixture to remove ice and snow and pointed out that the adhesion between the salt storage asphalt mixture and ice decreased. The adhesive force between the open-graded asphalt mixture and the ice is greater than that of the densely-graded asphalt mixture, and the adhesive force increases with the nominal maximum particle size. The salt storage asphalt mixture has good road performance, and its high-temperature performance and low-temperature performance are improved compared with ordinary asphalt mixture. Although moisture resistance is slightly decreased, it still meets the specification requirements. In the following year, Tan [22] developed a four-component slow-release complex salt filler. The carrier of the filler was optimized and the production process was determined according to the slow-release degree of the filler. The test of melting ice and snow shows that the pavement material mixed with slow-release complex salt filler has good de-icing and snow performance at the local temperature of −10 to 0 °C, and can reduce the adhesion of snow to the pavement at −20 to −10 °C. Yu [23] prepared a high-elasticity salt storage asphalt mixture using high-elasticity modified asphalt, and its high-temperature stability and low-temperature crack resistance were better than the SBS-modified asphalt mixture and salt storage asphalt mixture. The ice-breaking rate of high elastic salt storage asphalt mixture is 42% higher than that of ordinary SBS asphalt mixture, and it has the potential to remove an ice layer with 12 mm thickness. Guo [24] observed the microscopic morphology of the snow melting agent and studied the effect of the agent on the moisture resistance of the asphalt mixture. The porous structure on the surface of the de-icer is helpful for the release of NaCl, the main anti-condensation component. SMS can lower the freezing point to −2 °C. The salt solution in which the de-icing agent is dissolved is more likely to wet the aggregate, which reduces the moisture resistance of the asphalt mixture under the dual action of the dynamic water pressure and the salt solution. Wu [25] compared the storage and slow-release performance of six kinds of salt storage carriers for SMS and pointed out that the salt-storage carrier had little effect on the slow-release effect of SMS. The smaller the mass ratio of the salt storage carrier to the SMS, the better the snow melting effect of the pavement, but the worse the moisture resistance of the pavement at the same time. Considering the snow melting performance and mechanical properties of the asphalt mixture comprehensively, it is recommended that the mass ratio of SMS and carrier be 3.5:1.

At present, most of the research on salt storage pavement focuses on the performance of the asphalt mixture or melting ice and snow, which promotes the development of salt storage pavement technology to a certain extent. In salt storage pavement materials, SMS is mainly dispersed in asphalt, which makes SMS and asphalt generally exist in the form of asphalt mastic. Asphalt mastic is an important component of the asphalt mixture, and its performance directly affects the service of pavement [26,27,28,29]. However, there are few reports on the effect of SMS on the performance of asphalt mastic. Therefore, this paper uses two different modified asphalts, namely, SBS modified asphalt and HEA, to explore the effect of asphalt and SMS on the performance of asphalt mastic through traditional performance tests, temperature sweep tests, multiple stress creep and recovery tests, and bending beam rheological tests. This work can provide a reference for the research and application of salt storage asphalt pavement materials.

## 2. Materials and Methods

### 2.1. Materials

#### 2.1.1. Asphalt

The asphalt used in this study is SBS I-D, and its basic properties are shown in Table 1.

#### 2.1.2. Modifier

The mechanical properties of salt storage asphalt mixtures are often lost to varying degrees due to the addition of slow-release salt-storage materials, especially the loss of moisture resistance. Therefore, high elastic asphalt (HEA) was selected to improve the performance of the salt storage asphalt mixture. HEA is a modified asphalt made by adding plasticizers and cross-linking agents to SBS-modified asphalt. Compared with SBS modified asphalt, HEA has a higher elastic recovery rate, ductility, and softening point due to the addition of the high elastic agent, so HEA asphalt mixture also has better high-temperature performance, low-temperature performance, and fatigue performance.

**Figure 1:**
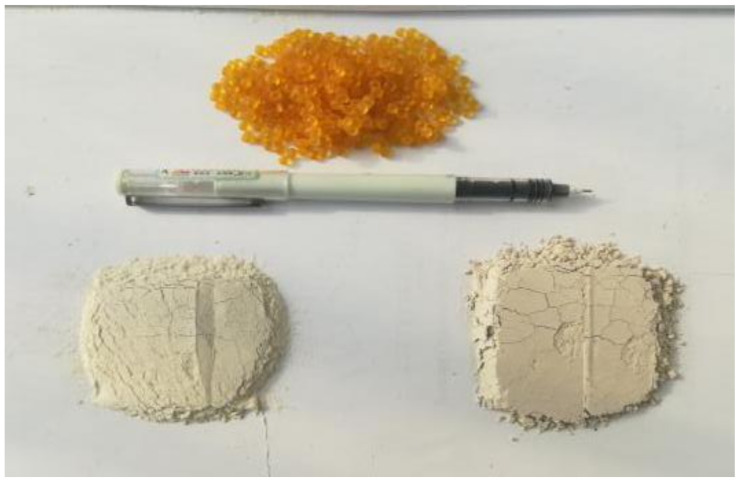
TPS modifier, Icebane, and limestone filler.

The high elastic agent used in this paper is TAFPACK-Super (TPS), and the appearance is orange-yellow transparent particles as shown in Figure 1. The main component of TPS is thermoplastic rubber, which is made with auxiliary component adhesive resin, and then mixed with other stabilizers. The basic properties of TPS are shown in Table 2.

#### 2.1.3. Filler

There are two kinds of fillers used in this paper, one is limestone filler and the other is SMS filler. The basic properties of limestone fillers are shown in Table 3. The SMS filler is Icebane, new ecological SMS, whose main chemical components include silicon dioxide, sodium chloride, calcium oxide, magnesium oxide, etc. Icebane is more than half of the effective snowmelt composition, and the chloride salts are adsorbed in the porous structure. In the rain and snow environment, the active ingredients can be gradually released to achieve the snow melting effect. The basic properties of Icebane are shown in Table 4. The comparison between Icebane and ordinary limestone filler is shown in Figure 1. The white powder particles on the left are Icebane, and the powder on the right is limestone filler.

### 2.2. Preparation of Modified Asphalt and Asphalt Mastic

#### 2.2.1. Preparation of HEA

A small amount of TPS high-elasticity modifier was added to the SBS modified asphalt at 175 °C several times. To better control the temperature and avoid asphalt aging, oil bath heating equipment is used to control the temperature throughout the process. Agitation was performed using an asphalt shearing machine at a shear rate of 2000 rpm. The addition ratio of high elastic agent and asphalt is 8:92. After the TPS was added to the SBS asphalt, it was kept at 2000 rpm for 10 min to make the modifier completely dissolve into the SBS modified asphalt. After that, the asphalt temperature was raised to 180 °C, and the asphalt was sheared for 35 min under the shearing speed of 3000 rpm to ensure a sufficient reaction between TPS and asphalt. The basic indicators of the HEA are shown in Table 5.

#### 2.2.2. Preparation of Salt Storage Asphalt Mastic

The amount of filler in the mixture is small. To maintain long-term snowmelt performance, a higher filler to asphalt ratio is required. However, an excessively large filler-to-asphalt ratio will lead to a reduction in free asphalt in the mastic, and the surface of some fillers cannot be covered by free asphalt. Some studies have pointed out that when the filler and asphalt ratio is 1.2:1, the mechanical properties of asphalt mastic can be maintained at a good level. Therefore, asphalt mastic was prepared with a filler to asphalt ratio of 1.2:1 [30]. In addition, the density of limestone filler is slightly higher than that of Icebane. If the SMS is added in an equal mass, the volume of the SMS should be greater than that of the limestone filler of equal mass. Therefore, Icebane is added to the asphalt matrix in the form of equal volume replacement to prepare salt storage asphalt mastic.

Due to the difference in density between asphalt and filler, the two are prone to segregation, which affects the performance of asphalt mastic. To this end, the glass rod is used for continuous stirring to ensure the uniformity of the asphalt mastic. The preparation process is detailed as follows:

(1) Both the limestone filler and the salt storage material were passed through a 0.075 mm sieve, and the bottom part of the sieve was taken for testing. The two fillers were placed in a dry and ventilated place, and the fillers were dried and ground before the test to avoid agglomeration affecting the accuracy of the test.
(2) The pitch was heated to 175 °C to ensure that the pitch was picked out by the glass rod drips in the form of droplets. The asphalt, preheated to the same temperature as the asphalt in portions, was added. The glass rod was kept agitated to prevent the filler from sticking to the container walls. The heating temperature and stirring time were strictly controlled to avoid asphalt aging.
(3) The different types of asphalt mastics were named SBS00, SBS50, HEA50, SBS75, HEA75, SBS100, and HEA100 according to the proportion of Icebane replacing limestone filler. For example, SBS00 means that the replacement rate of limestone filler is 0%, and the base asphalt is SBS-modified asphalt.

### 2.3. Testing Methods

#### 2.3.1. Testing of Three Major Indicators: Penetration, Softening Point, and Ductility

To compare the effects of asphalt and SMS on the basic properties of asphalt mastic, the penetration, softening point, and ductility of salt storage asphalt mastic were tested with regard to the “Standard Test Methods of asphalt and Bituminous Mixtures for Highway Engineering (JTG E20-2011)” [31]. Penetration reflects the consistency and hardness of asphalt. Generally speaking, the smaller the penetration, the better the high-temperature rheological resistance of asphalt. The softening point also reflects the high-temperature resistance of asphalt. The higher the softening point, the stronger the thermal storage capacity of the asphalt and the better the thermal stability. Ductility reflects the low-temperature deformation properties of asphalt; that is, the plastic properties of asphalt. The higher the ductility, the better the plasticity of the asphalt and the better the low-temperature crack resistance. Sample information is shown in Table 6.

#### 2.3.2. Brookfield Viscosity Test

The viscosity-temperature characteristics of asphalt mastic not only affect the mixing and compaction process of the mixture but are also closely related to the high and low-temperature performance and durability of the mixture. For the salt storage asphalt mixture, the selection of the engineering mixing temperature and the rolling temperature should refer to the viscosity of the asphalt mastic. If the viscosity of the asphalt mastic formed by Icebane and asphalt is not fully studied, it will increase the difficulty of mixing and compacting the asphalt mixture. For example, the poor adhesion between Icebane and asphalt will increase the fluidity of the mixture, cause leakage during the transportation and paving stages of the mixture, and endanger the flatness after compaction. Therefore, to explore the advantages and disadvantages of salt storage asphalt mixture and ordinary mixture, it is necessary to study the viscosity–temperature characteristics of asphalt mastic. Referring to the specification [31], a 28# rotor was selected to test the Brookfield viscosity of asphalt mastic with different substitution rates of SMS.

#### 2.3.3. Temperature Sweep Test

By studying the viscoelastic properties of salt storage asphalt mastic, the effect of SMS on the performance of salt storage asphalt mixture can be further understood. The SHRP specification uses dynamic shear rheological testing as a measure of asphalt performance. The mechanical properties of the salt storage asphalt mastic are analyzed by the complex modulus, phase angle, and other indicators, to make a more scientific and reasonable evaluation of the characteristics of the mixture. In this paper, the DHR-1 dynamic shear rheometer produced by TA company in the United States is used for testing. During the test, it should be noted that the distance between the upper and lower parallel plate fixtures is selected to be 1 mm, which is suitable for the test above 30 °C, and the size of the matching parallel plate is selected to be 25 mm [32,33].

When the temperature sweep test was performed on the salt storage asphalt mastic, the temperature sweep range of was set to 40–80 °C. The temperature interval was 5 °C, the strain was set to 10%, and the angular frequency parameter was set to 10 rad/s. The storage modulus *G’*, complex modulus G*, phase angle *δ*, rutting factor G*/sin*δ*, and *Z* are used as indicators to evaluate the high-temperature performance of salt storage asphalt mastic.

#### 2.3.4. Multiple Stress Creep and Recovery Test

For modified asphalt, simply using the temperature sweep test to evaluate the high-temperature performance is insufficient [29]. AASHTO TP70 proposes to use multiple stress creep and recovery tests to evaluate the high-temperature performance of asphalt. Therefore, according to the repeated creep test procedure, repeated loading tests were carried out on the asphalt mastic under two stress levels of 0.1 and 3.2 kPa at 58, 64, and 70 °C, to simulate the repeated creep effect of light and heavy traffic loads on the asphalt pavement. In each loading cycle, the loading time is 1 s and the recovery time is 9 s. The repetitions for each test are 10 times and the total time is 200 s.

As two indicators for evaluating the repeated creep characteristics of asphalt, the creep recovery rate *R* and the non-recoverable creep compliance *J_nr_* are calculated according to Equation (1) and Equation (2), respectively. The higher the creep recovery rate, the smaller the non-recoverable creep compliance, indicating that the asphalt has sufficient deformation recovery ability under repeated stress.

(1) $$R=\frac{\gamma_{p}-\gamma_{nr}}{\gamma_{p}-\gamma_{0}}\times 100\%$$

(2) $$J_{nr}=\frac{\gamma_{nr}-\gamma_{0}}{\tau }$$

where *γ_0_* is the initial strain; *γ_p_* is the peak strain; *γ_nr_* is the irrecoverable strain; and *τ* is the creep stress.

#### 2.3.5. Bending Beam Rheological Test

In the SHRP program of the United States, it is proposed to use the bending beam rheological test to evaluate the low-temperature cracking resistance of asphalt. Two parameters, the modulus of creep stiffness *S* and the creep slope *m*, can be obtained through the bending beam rheological test. The smaller the stiffness modulus and the larger the creep rate, the better flexibility and low-temperature crack resistance of the asphalt. This test method is more accurate and is also widely used for the performance grading of asphalt. Since the asphalt pavement will be affected by the coupling of traffic load and temperature during the service period, the stiffness modulus of the asphalt will increase after aging, and the asphalt pavement will be more prone to cracks. Test pieces with standard dimensions of 102 × 12.5 × 6.25 mm^3^ were made by pouring asphalt mastic into a special mold. The specimens were then placed in anhydrous ethanol at the specified temperature for incubation. The test temperature is selected as −24, −18, and −12 °C. During the test, the mid-span load of the trabecular was 980 mN and the duration was 240 s. The creep stiffness *S*(*t*) can be calculated by Equation (3).

(3) $$S(t)=\frac{PL^{3}}{4bh^{3}\delta (t)}$$

where *L* is the distance between the fulcrums, m; *P* is the applied load, kN; *h* is the thickness of the specimen, m; *b* is the width of the specimen, m; *δ*(*t*) is the maximum deflection, m; and *S*(*t*) is the Creep stiffness at time *t*, MPa.

## 3. Results and Discussion

### 3.1. Penetration, Softening Point, and Ductility

**Figure 2:**
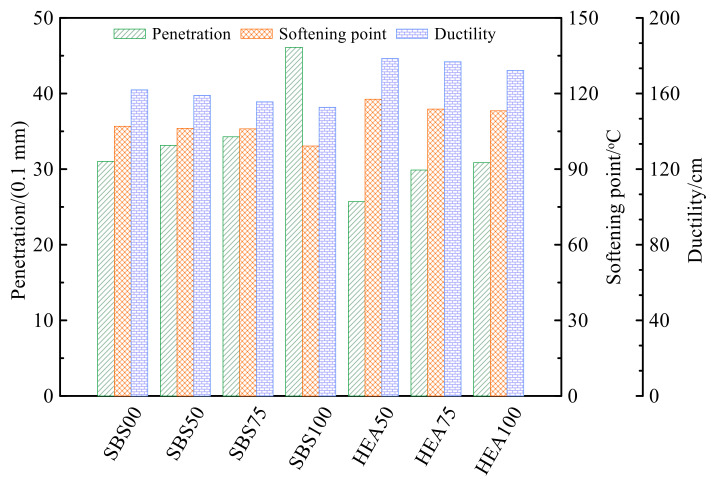
Basic properties of asphalt mastic.

Penetration, softening point, and ductility are used to characterize the basic properties of asphalt. Generally speaking, the greater the penetration, the smaller the asphalt consistency, the weaker its rutting resistance, and the better the low-temperature toughness. The higher the softening point, the more resistance of the asphalt to high temperatures. The higher the ductility, the less likely the asphalt will break at low temperatures. With the help of these three indicators, the performance of salt storage asphalt mastic is preliminarily evaluated. Figure 2 shows the penetration, softening point, and ductility of the asphalt mastics of the HEA group and the SBS group. Overall, the HEA asphalt mastic has smaller penetration, and a higher softening point and ductility than the SBS asphalt mastic, indicating that the HEA group has excellent low-temperature and high-temperature performance. This may be because the high elastic modifier TPS forms an inter-crosslinked network structure in the asphalt. This network structure binds the thermal motion of asphalt molecules at room temperature and high temperature and increases the toughness of asphalt at low temperature [34,35,36]. Therefore, the asphalt mastic of the HEA group has better high-temperature and low-temperature performance than that of the SBS group [14,37].

Figure 2 shows the effect of SMS on penetration, softening point, and ductility of asphalt mastic. In terms of penetration, in both the SBS group and the HEA group of salt storage asphalt mastic, the penetration increased with the replacement amount of SMS. This indicates that the addition of SMS softened the asphalt mastic. The SMS replaces the limestone filler with the equal volume replacement method. The particle size of the SMS is generally larger than that of the limestone filler, so the number of SMS particles may be less than that of the limestone filler. SMS filler is coarser, the proportion of structural asphalt in asphalt mastic decreases, and the proportion of free asphalt increases [20,38,39]. Smaller salt particles can enhance asphalt mastic performance [37]. Therefore, as the content of SMS increases, the penetration also gradually increases. Differences in the properties of the two fillers may also be responsible for the increased penetration. The main component of SMS is chloride salt, which has weaker interaction with asphalt than limestone fillers [14,40,41]. In addition, the poor adhesion of SMS to asphalt changes the continuous state of asphalt, and the reduced viscosity of asphalt mastic may also be the reason for the increased penetration [42,43]. In terms of softening point, the replacement amount of SMS increased, and the softening point of the two groups of salt storage asphalt mastics decreased. The decrease in softening point indicates that the heat required for asphalt mastic to obtain flow deformation ability decreases; that is, the energy required for asphalt molecules to overcome intermolecular forces decreases [36,44]. The reduction in the interaction between filler and asphalt due to the reduction in structural asphalt can also be explained as the lowering of the softening point. In terms of ductility, the ductility of the salt-storing asphalt mastic in the SBS group and the HEA group has a decreasing trend with the increase in the SMS content, but the decrease is not obvious. When the content of SMS increases, the content of free asphalt increases, and the low-temperature ductility of asphalt mastic should increase. However, the ductility is reduced here, probably because the reduction in structural asphalt leads to a reduction in the asphalt bond strength. The reduction in the ductility of asphalt by SMS was also demonstrated in the literature [45]. The salt-storing asphalt mastic is in a discontinuous state, and the shrinkage coefficients of SMS and asphalt are different. The higher the content of SMS, the worse the interface continuity of the asphalt mastic, the worse the activity of the mastic, and the lower the ductility [45,46].

From the three basic index results of penetration, softening point, and ductility, it can be preliminarily concluded that HEA has better high-temperature and low-temperature performance than SBS-modified asphalt. SMS has adverse effects on the high-temperature and low-temperature properties of asphalt mastic. The basic performance of HEA100 is slightly better than that of SBS00 without SMS, so adding TPS can make up for the negative impact of SMS on asphalt mastic to a certain extent. Nevertheless, based on these three indicators, the performance of asphalt mastic can only be preliminarily evaluated. The influence of asphalt type and SMS on the performance of asphalt mastic requires further research on viscosity and rheological properties.

### 3.2. Brookfield Viscosity

**Figure 3:**
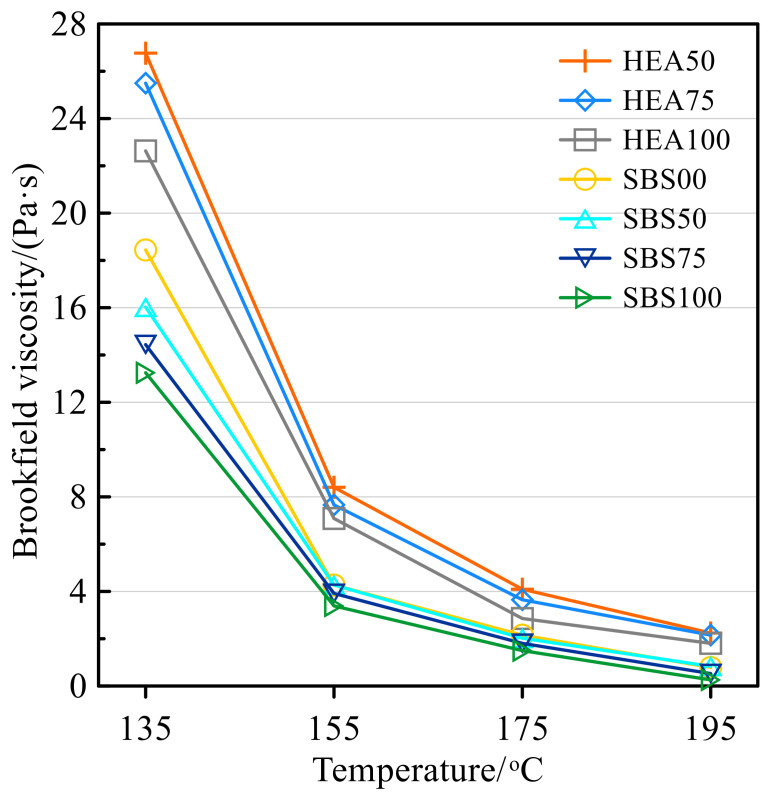
The viscosity of asphalt mastic at different temperatures.

In Figure 3, the viscosity of asphalt mastic shows a decreasing trend with temperature. For example, at 135 °C, the viscosity of HEA100 is 3.15, 7.28, and 11.94 times that of 155, 175, and 195 °C, respectively. This is because the increase in temperature accelerates the thermal motion of the asphalt molecules in the asphalt mastic, making the asphalt flow more easily [36,47]. Nonetheless, the higher the temperature, the smaller the viscosity difference between the individual asphalt mastics. The asphalt mastic of the HEA group showed higher viscosity than that of the SBS group at all four temperatures. This may be because the interaction of TPS and SBS-modified asphalt increases the content of macromolecules, making it difficult for HEA mastic to flow [47]. Furthermore, the network structure formed after TPS swelling hinders the thermal motion of asphalt molecules [34,35,36]. Whether it is HEA group or SBS group salt storage asphalt mastic, the higher the replacement rate of Icebane, the lower the viscosity of the asphalt mastic. Taking the HEA group as an example, when the content of Icebane in HEA is 75% and 100%, their viscosity at 135 °C is 4.47% and 11.32% lower than that of HEA50, respectively. The decrease in viscosity with the content of SMS may be because the bond between SMS and asphalt is weaker than that of limestone fillers [14,40,41]. Furthermore, the reduction in structural asphalt may also be one of the factors leading to the reduction in asphalt viscosity [19,20,39]. In a word, there are two rules for the viscosity of salt storage asphalt mastic, one is that the viscosity of the HEA group is higher than that of the SBS group, and the other is that limestone filler contributes more to asphalt viscosity than Icebane.

### 3.3. Analysis of Temperature Sweep Test Results

#### 3.3.1. Storage Modulus, Phase Angle, Complex Modulus, and Rutting Factor

**Figure 4:**
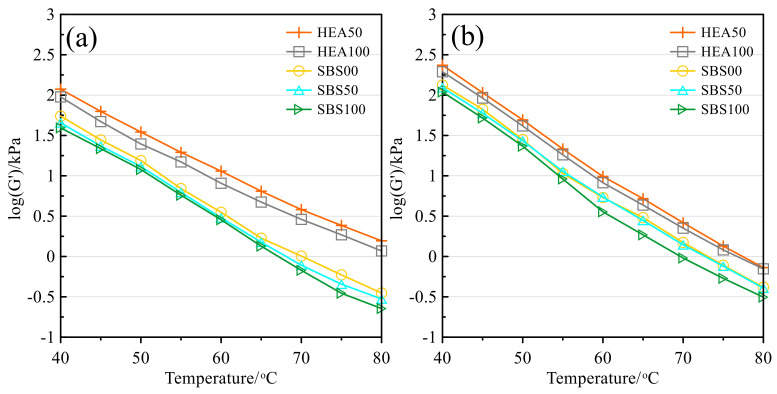
Storage modulus: (**a**) Before TFOT; (**b**) After TFOT.

As shown in Figure 4, the storage modulus of asphalt mastic shows a decreasing trend with increasing temperature. This is because the plasticity of the asphalt increases and the elastic properties decrease as the temperature increases [44,47]. The storage modulus of asphalt mastic becomes larger after aging because the light components are gradually transformed into resins and asphaltenes after asphalt aging [36,44]. The heavy component has a larger molecular weight, small molecular distance, strong interaction force, and good elastic recovery performance [36,44,47,48]. Overall, low temperature and aging will increase the elastic components in asphalt, which is conducive to the elastic recovery of mastic. In addition, the content of Icebane also affects the elastic component in the asphalt. The higher the replacement rate of SMS, the worse the elastic recovery of asphalt mastic, which was confirmed in both the SBS group and the HEA group. This may be because the increase in SMS reduces the structural asphalt in asphalt mastic, while structural asphalt is beneficial to improving the elastic recovery performance of asphalt mastic [20,39]. The storage modulus of the asphalt mastic in the HEA group was higher than that of the SBS group. This is because TPS directly increases the elastic component in SBS-modified asphalt as a high elastic agent. In addition, the storage modulus of HEA100 with the highest content of SMS in the HEA group was even higher than that of SBS00 without SMS. This indicates that the performance of the asphalt could compensate for the unfavorable effect of the SMS filler on the elastic deformation capacity of the mastic.

**Figure 5:**
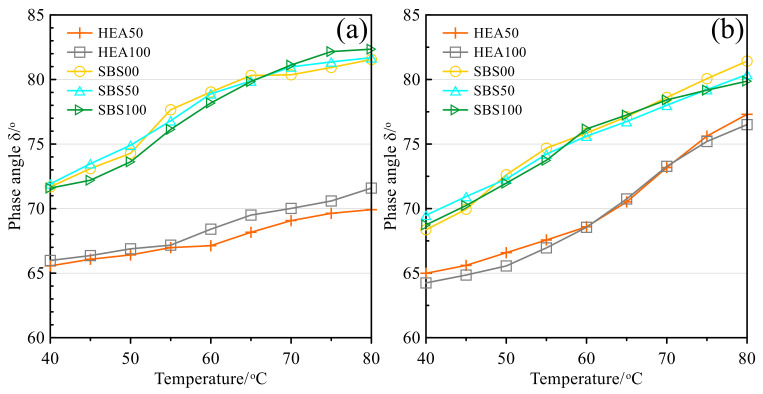
Phase angle: (**a**) Before TFOT; (**b**) After TFOT.

Figure 5 shows the phase angle of the asphalt mastic before and after aging as a function of temperature. As an index of asphalt viscoelasticity, the phase angle can reflect the proportion of viscoelastic components in asphalt [36]. As the temperature increases, the phase angle of the asphalt mastic increases gradually, because as the temperature increases, the viscous component in the asphalt increases, while the elastic component decreases [49]. Since TPS increases the elastic component in the SBS-modified asphalt, the phase angle of the HEA asphalt mastic is much smaller than that of the SBS group [50]. The phase angle decreases slightly after the asphalt is aged, mainly because the asphalt becomes brittle and hard after aging, the elastic component increases, and the viscous component decreases. As for the influence of the SMS on the phase angle, since the phase angle curves of different amounts of SMS in the two groups of asphalt mastic intersect with each other, the influence of the content of Icebane on the viscoelastic component proportion of asphalt mastic is relatively complex [49,51,52].

**Figure 6:**
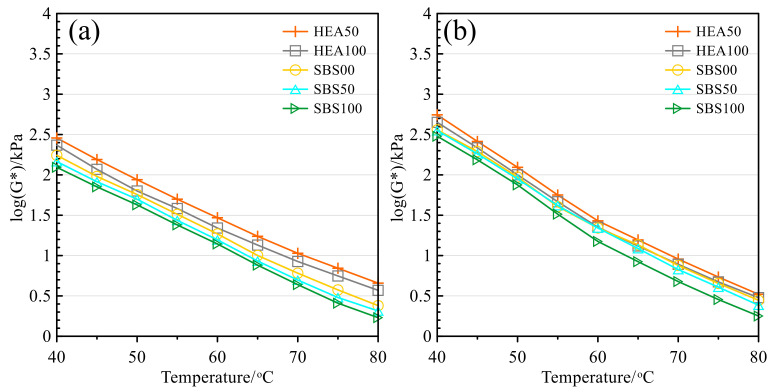
Complex modulus: (**a**) Before TFOT; (**b**) After TFOT.

**Figure 7:**
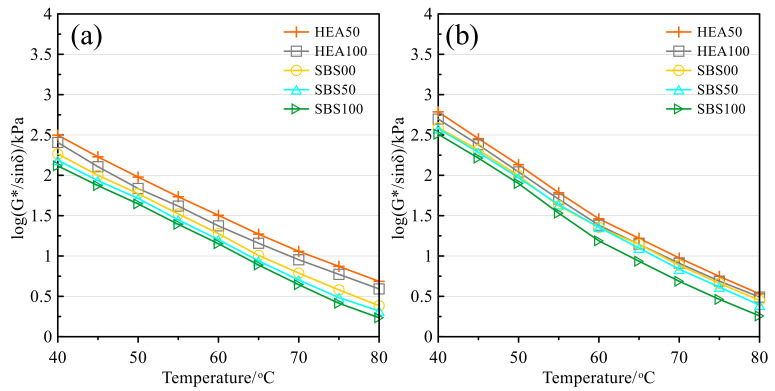
Rutting factor: (**a**) Before TFOT; (**b**) After TFOT.

Figure 6 and Figure 7 are the changes in the complex modulus and rutting factor with temperature before and after the aging of asphalt mastic, respectively. These two indicators can be used to measure the rheology of asphalt mastic at high temperatures [36,53]. The complex modulus and rutting factor have similar trends, so the high-temperature performance of salt storage asphalt mastic is analyzed by taking the rutting factor as an example. It can be seen from Figure 7 that with the increase in temperature, the rutting factor shows a decreasing trend, which indicates the ability of asphalt to resist high-temperature rutting decreases. This is because the intermolecular force weakens at high temperatures and the molecular motion of asphalt accelerates [36,49]. After aging, the rutting factor increases, and aging contributes to improved high-temperature performance [44,49,54]. Regardless of the test temperature, the order of rutting factors from high to low is HEA50, HEA100, SBS00, SBS50, and SBS100. Therefore, the high-temperature performance of salt storage asphalt mastic before and after aging will be affected by the asphalt type and the Icebane content. The inclusion of TPS in HEA can increase the rutting resistance of the asphalt mastic, while the higher the SMS content, the smaller the deformation resistance. The high-temperature rheological properties of salt-storing asphalt mastic are consistent with Section 3.1 and Section 3.2.

#### 3.3.2. Viscoelastic Index Z

**Figure 8:**
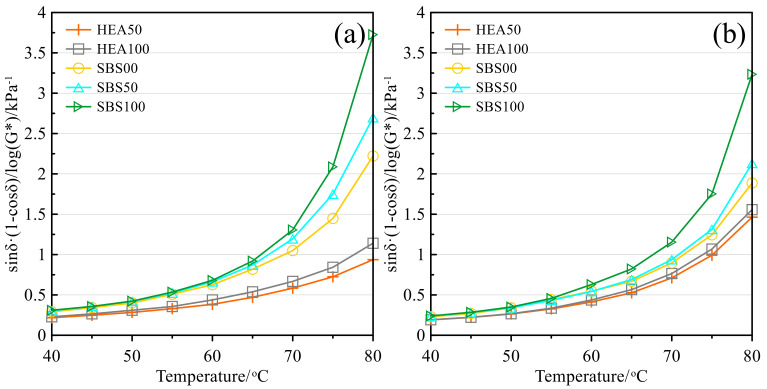
Viscoelastic index Z: (**a**) Before TFOT; (**b**) After TFOT.

Similar to the rutting factor, the viscoelastic index Z [Z=sinδ·(1-cosδ)/log(G*)] can also reflect the rutting resistance of asphalt mastic [36,55]. The smaller the Z value, the stronger the high-temperature performance of asphalt mastic. Figure 8 shows the viscoelastic index Z of the salt storage asphalt mastic before and after aging. The index Z of the asphalt mastic in the HEA group and the SBS group increases with the temperature, the viscosity of the mastic decreases, and the mastic gradually changes to a viscous-fluid state [47]. Under the same amount of SMS, the Z value of the HEA group was always lower than that of the SBS group. This shows that the high elastic agent increases the rutting resistance of the asphalt mastic. The Z value increases with the content of SMS, which reflects that SMS has a hindering effect on the high-temperature performance of salt-storing asphalt mastic. Comparing the Z values of SBS00 and HEA100, it can be concluded that the improvement effect of the high elastic agent on the high-temperature performance of asphalt mastic is much higher than the negative effect of SMS on the high-temperature performance. This analysis is consistent with the previous analysis. The Z value of the aged asphalt mastic becomes smaller because the asphalt becomes hard and brittle after aging [36,56].

### 3.4. Analysis of MSCR Test Results

#### 3.4.1. Accumulated Strain

**Figure 9:**
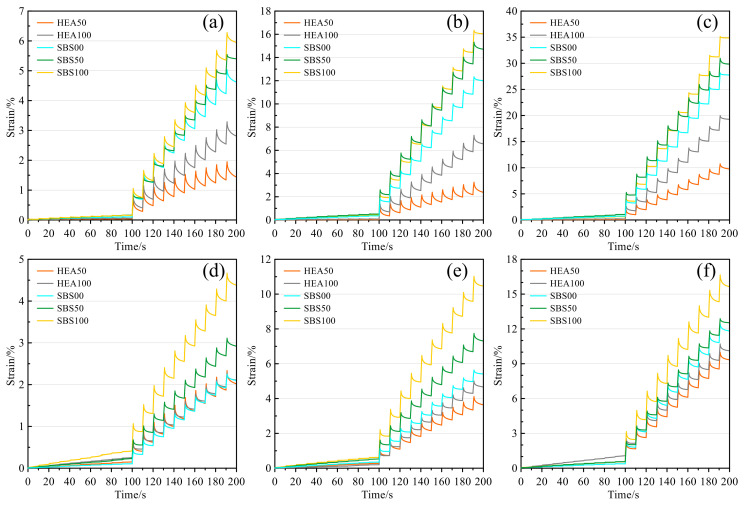
Strain curve: (**a**) 58 °C before TFOT; (**b**) 64 °C before TFOT; (**c**) 70 °C before TFOT; (**d**) 58 °C after TFOT; (**e**) 64 °C after TFOT; (**f**) 70 °C after TFOT.

Rutting is the accumulation of pavement deformation under repeated traffic loads. The cumulative strain of asphalt mastic can also characterize the service performance of the pavement. The higher the value, the worse the rutting resistance of the pavement [57]. The strain of asphalt mastic under cyclic loading is shown in Figure 9. The higher the temperature, the higher the strain of the asphalt mastic. The elevated temperature enhances the thermal motion of the asphalt molecules, while the elastic component in the asphalt mastic gradually transforms into the viscous component [36]. Short-term aging will make the asphalt mastic hard and brittle, thereby reducing the strain of the mastic. In addition, with the increase in the amount of SMS, the strain of asphalt mastic under load also increases gradually. The interaction between SMS and asphalt is weak, and the addition of SMS may increase the free asphalt content in asphalt mastic, so SMS promotes the deformation of asphalt mastic compared to limestone filler [20,40,41]. Under each stress condition, the order of strain from high to low is SBS100 > SBS50 > SBS00 > HEA100 > HEA50. The cumulative strain of the HEA group was smaller than that of the SBS group. The high elastic properties of HEA can well reduce the strain of mastic under creep conditions, which may be due to the network structure formed after the swelling of the high elastic modifier in the asphalt, which limits the deformation of the asphalt mastic [36]. Except for Figure 9d, the cumulative deformation of HEA100 is always lower than that of SBS00. Even in Figure 8b, the cumulative strain of HEA100 is not higher than that of SBS00. Therefore, the use of SMS in combination with high elastic agents not only provides snow melting potential to the pavement but also improves the rutting resistance.

#### 3.4.2. Creep Compliance and Creep Recovery

**Figure 10:**
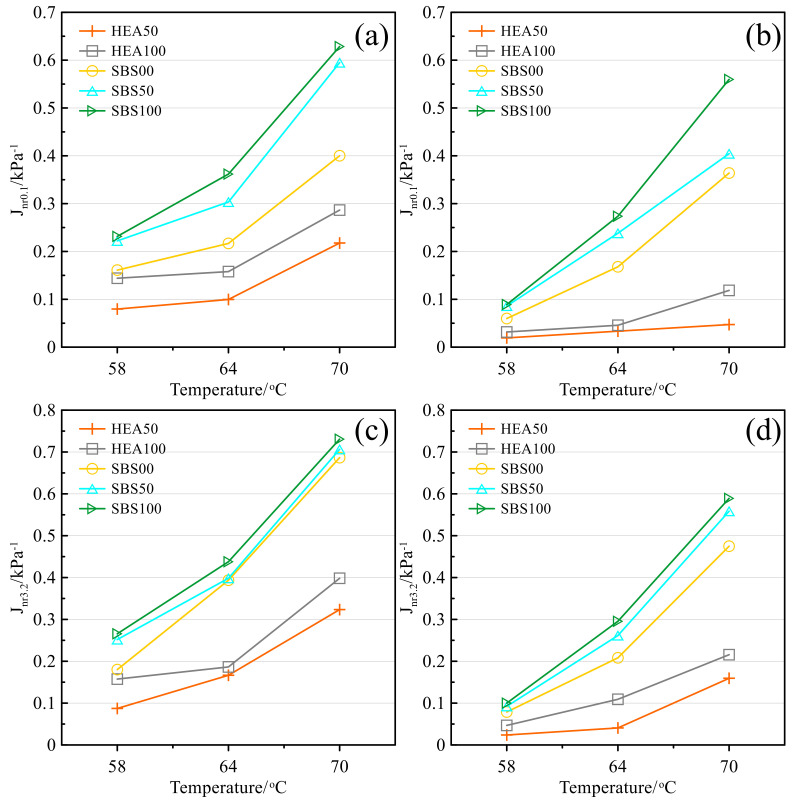
Creep compliance: (**a**) 0.1 kPa before TFOT; (**b**) 0.1 kPa after TFOT; (**c**) 3.2 kPa before TFOT; (**d**) 3.2 kPa after TFOT.

**Figure 11:**
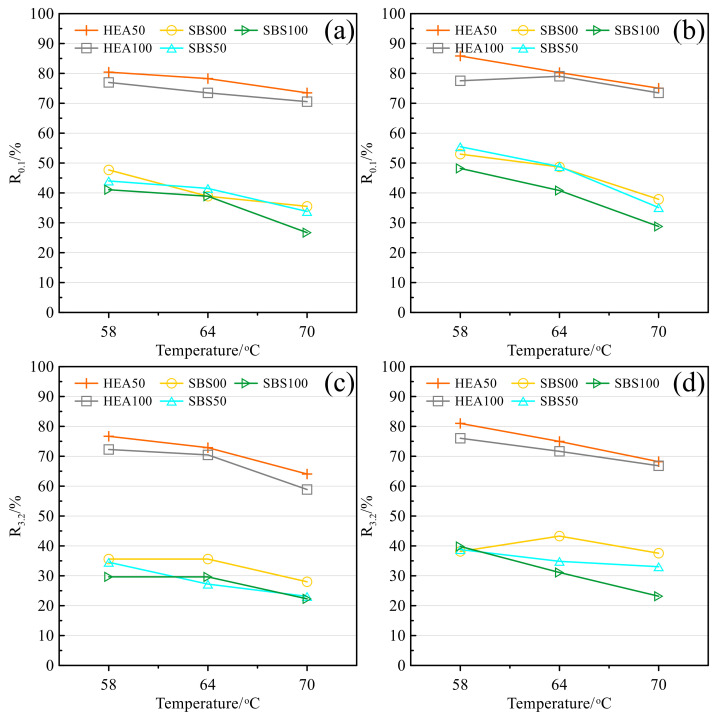
Recovery rate: (**a**) 0.1 kPa before TFOT; (**b**) 0.1 kPa after TFOT; (**c**) 3.2 kPa before TFOT; (**d**) 3.2 kPa after TFOT.

Creep compliance reflects the high-temperature deformation capacity. The higher the value, the easier it is for the asphalt mastic to creep at high temperatures. The creep compliance of different salt storage asphalt mastics before and after aging is shown in Figure 10. In Figure 9a, the creep compliance increases with temperature because high temperature reduces the viscosity of the mastic and enhances the mobility of asphalt molecules [36]. The creep compliance of the HEA group is lower than that of the SBS group, indicating that HEA is more resistant to high-temperature creep. The creep compliance increases with the content of SMS, so SMS is not beneficial to the creep resistance of asphalt mastic. This may be due to the reduction in structural asphalt in asphalt mastic by SMSs [20,39]. Although limestone filler can give salt storage asphalt mastic better high-temperature performance, with the help of the high elastic agent, the creep compliance of HEA100 without limestone filler is still stronger than SBS00 without SMS. The influence of asphalt type on the high-temperature performance of the mastic is higher than that of SMS. The trend of creep compliance in Figure 9b–d is similar to that in Figure 9a. Comparing the four subplots in Figure 9, it can be seen that the aging effect can reduce the creep compliance, which is due to the reduction in light components in the aged asphalt mastic and the hardening of the asphalt mastic [36,56]. In addition, increasing the stress level will increase the creep compliance of the asphalt mastic, which is related to the increase in the cumulative deformation of the asphalt mastic caused by increasing the stress in Figure 9. Both increasing stress and increasing temperature will increase creep compliance, indicating that the effect of high load and high temperature has the same effect on the creep of asphalt mastic [57]. This equivalent effect is also confirmed in Figure 11.

Creep recovery is the opposite of creep compliance and reflects the ability to resist high-temperature deformation. The higher the value, the better the deformation resistance of asphalt mastic [58]. The creep recovery of different types of salt storage asphalt mastics before and after aging is shown in Figure 11. In Figure 11a, the increase in temperature will lead to a decrease in the recovery of asphalt mastics, indicating that the increasing temperature will increase the viscous component of asphalt and reduce the elastic component, so the deformation recovery ability will decrease accordingly. The recovery rate of HEA50 and HEA100 salt storage asphalt mastics is much higher than other types of mastics. The recovery rate of the two groups of asphalt mastics decreased with the content of SMS, which was opposite to the creep compliance, but the reason was the same. The recovery rate of HEA100 was higher than that of SBS00. After aging, the *R_3.2_* index of HEA50 and HEA100 were 30.7% and 29.4% higher than that of SBS00 at 70 °C, respectively. Comparing Figure 11a–d, it is found that when the stress level increases from 0.1 to 3.2 kPa, the recovery rate decreases, indicating that the asphalt material can only exert a good elastic recovery ability within a certain external load [58]. So, this may be one of the reasons for limiting overloading. After aging, the recovery rate of all types of asphalt mastic increased significantly, because aging resulted in the transformation of light components into resins or asphaltenes. The heavy components have strong intermolecular forces, strong resistance to deformation, and good recovery performance [36].

### 3.5. Analysis of BBR Test Results

#### 3.5.1. Creep Stiffness and Creep Rate

Creep stiffness can reflect the creep resistance of asphalt mastic at low temperature. The creep stiffness of different salt storage asphalt mastics is shown in Figure 11a. The creep stiffness increases with the decrease in temperature, which is due to the hardening and brittleness of the asphalt mastic [57]. For example, the creep stiffness of HEA100 increased by 181.4% after the temperature was lowered from −12 to −18 °C. After the temperature was decreased from −18 to −24 °C, the creep stiffness increased by 99.2%. Comparing the creep stiffness of the HEA group and the SBS group, the creep stiffness of the HEA group is smaller than that of the SBS group. This shows that TPS not only increases the high-temperature performance of asphalt mastic but also improves the low-temperature performance [14,17,50]. The improvement in low-temperature performance may be due to the reinforcement and toughening effect of TPS in asphalt, which is consistent with the change in ductility. In addition, the creep stiffness was also affected by the SMS and the creep stiffness increased with the SMS content. As mentioned earlier, SMS increases the free asphalt content in the bituminous mastic. Generally speaking, as the free asphalt content increases, the asphalt mastic has a stronger low-temperature deformation ability and lower creep stiffness [59]. In this case, however, the creep stiffness increases with the SMS content. This may be because the SMS is distributed in the asphalt in a point-like manner, and with the increase in SMS, the interfacial phase increases, which causes the asphalt to become hard and brittle [40,60]. HEA decreased creep stiffness and SMS increased creep stiffness. Overall, the effect of HEA is more significant, which may be the reason why the creep compliance of HEA100 is lower than that of SBS00.

**Figure 12:**
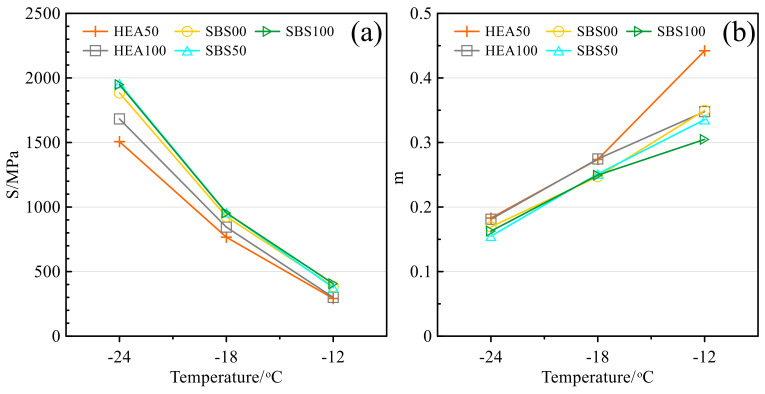
Low-temperature creep of asphalt mastic: (**a**) Creep stiffness. (**b**) Creep rate.

The creep rate reflects the ability of asphalt mastic to generate creep. The larger the value, the better the low-temperature deformation ability of asphalt mastic, and the less likely brittle fracture occurs [44,49,61]. The creep rates of different asphalt mastics are shown in Figure 12b. The creep rate of asphalt mastic increases with temperature, which is opposite to the trend of creep stiffness [57]. The m value of the HEA group was higher than that of the SBS group, indicating that the low-temperature performance of the HEA group was excellent. When the temperature is −12 °C, the effect of SMS content on creep rate is opposite to the *S* value. However, when the temperature is −18 °C and −24 °C, the effect of SMS on m is not obvious.

#### 3.5.2. m/S

**Figure 13:**
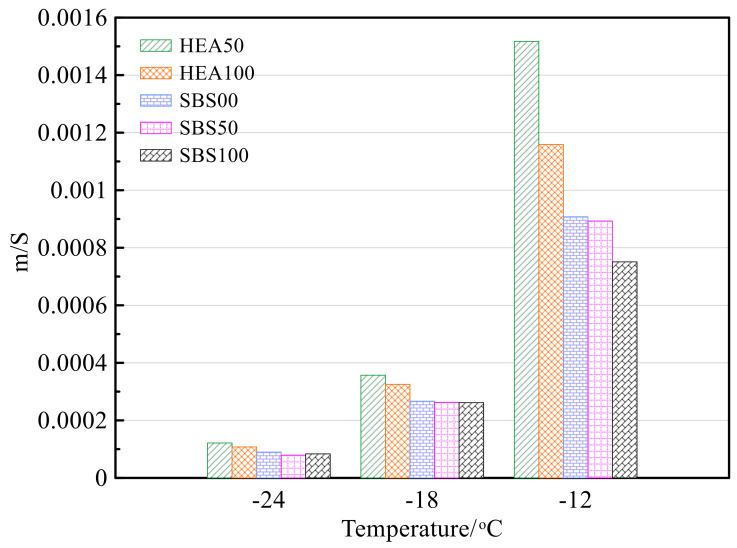
m/S of asphalt mastic.

The S value is sensitive to the SMS, while the m value changes significantly with the SMS content only at −12 °C. Other studies have pointed out that there is a contradiction between the S value and the m value [36,47,62]. Using m/S can more accurately characterize the performance of salt storage asphalt mastic at low temperature [36,63]. The larger the m/S value, the better the flexibility of the asphalt mastic, which is beneficial to the low-temperature performance. The m/S of different salt storage asphalt mastics is shown in Figure 13. With the increase in the SMS content, the m/S value decreased, indicating that SMS reduced the low-temperature performance of asphalt mastic. This is consistent with the reduction in the ductility of asphalt mastic by SMS. As the temperature decreases, the m/S value decreases significantly, and the brittleness of asphalt mastic gradually manifests. The m/S value of HEA100 is also higher than that of SBS00, so although SMS has a negative effect on low-temperature performance, this negative effect can be completely covered by HEA.

## 4. Conclusions

The addition of slow-release SMS affects the performance of the salt storage asphalt mixture. However, the slow-release SMS mainly exists in the form of asphalt mastic in the asphalt mixture. Studying the effect of SMS on the performance of asphalt mastic has potential significance for improving the mechanical properties of a salt-storing asphalt mixture. For this reason, different types of salt storage asphalt mastic were prepared using SBS-modified asphalt and HEA as the asphalt matrix. The effects of asphalt matrix and SMS on the properties of asphalt mastic were analyzed through basic physical property tests and rheological property tests. The main conclusions are as follows:

(1) The routine test of asphalt mastic points out that the performance of HEA asphalt mastic is generally better than that of SBS. HEA asphalt mastic has lower penetration, higher softening point, ductility, and viscosity. With the increase in SMS content, the penetration of asphalt mastic increases, and the softening point and ductility decrease. The higher the SMS content, the lower the viscosity of the asphalt mastic.
(2) The changes in the *G′*, G*, rutting factor, and *Z* show that the content of SMS in asphalt will affect the stiffness and deformation properties of asphalt mastic. The higher the replacement rate of SMS, the less conducive to the elastic recovery performance of asphalt mastic. The phase angle is affected by SMS, but not significantly. The high-temperature performance of the asphalt mastic of the HEA group is better than that of the SBS group.
(3) MSCR results show that the addition of SMS increases the deformation of asphalt mastic under load. Under each stress condition, the order of the various asphalt mastic strains is SBS100 > SBS50 > SBS00 > HEA100 > HEA50. The high elasticity of HEA can well reduce the creep of salt storage asphalt mastic. The gain effect of TPS on creep recovery can compensate for the negative effect of SMS.
(4) The *J_nr_* of the salt storage asphalt mastic increased with the content of SMS, while the *R* was the opposite. Although SMS hinders the high-temperature performance of asphalt mastic, HEA100 has better high-temperature performance than SBS00 with the help of high elastic agent TPS. The addition of TPS can make up for the adverse effect of SMS on the high-temperature performance of asphalt mastic.
(5) The creep stiffness increases with the content of SMS, and SMS reduces the creep rate and *m*/*S* of asphalt mastic. The larger the content of SMS, the worse the low-temperature crack resistance of the mastic. TPS can reduce the weakening effect of SMS on the low-temperature toughness of asphalt mastic.

## Figures and Tables

**Table 1:** Basic technical indexes of SBS I-D asphalt.

Indexes	Unit	Test Result	Standard	Test Method
Penetration (100 g, 5 s, 25 °C)	0.1 mm	54	40–60	T0604-2011
Ductility (5 °C, 5 cm/min)	cm	31	≥20	T0605-2011
Softening Point	°C	80	≥60	T0606-2011
Dynamic viscosity (135 °C)	Pa·s	1.773	≤3	T0620-2011
Elastic recovery (25 °C)	%	90	≥75	T0662-2000
Residues after TFOT				
Mass change	%	−0.213	±1.0	T0609-2011
Penetration ratio (25 °C)	%	70.5	≥65	T0604-2011
Ductility (5 °C, 5 cm/min)	cm	16	≥15	T0605-2011

**Table 2:** Basic properties of TPS.

Particle Size/mm	Color	Relative Density	Water Absorption Rate/%	Melting Point/°C
2–4	Yellow	0.96	<1	170

**Table 3:** Basic properties of limestone fillers.

Indexes	Test Result	Standard	Test Method
Moisture content (%)	0.2	≤1.0	T0103-1993
Apparent relative density	2.762	≥2.5	T0352-2000
Gross bulk relative density	2.667	/	T0352-2000
Hydrophilic coefficient	0.73	<1	T0353-2000
Plasticity index (%)	2.7	<4	T0354-2000
Appearance	Qualified	No agglomeration	T0355-2000

**Table 4:** Basic properties of Icebane.

Indexes	Test Result	Standard	Test Method
Appearance	White powder	No agglomeration	T0355-2000
Moisture content (%)	0.2	≤0.5	T0103-1993
Apparent relative density	2.170	/	T0352-2000
Gross bulk relative density	2.136	/	T0352-2000
Salt content (%)	56	50 ± 10	/
pH	8.3	8–8.5	/

**Table 5:** Basic indicators of HEA.

Indexes	Unit	Test Result	Standard	Test Method
Penetration (100 g, 5 s, 25 °C)	0.1 mm	49	40–60	T0604-2011
Ductility (5 °C, 5 cm/min)	cm	55	≥20	T0605-2011
Softening Point	°C	94	≥60	T0606-2011
Dynamic viscosity (135 °C)	Pa·s	1.861	≤3	T0620-2011
Elastic recovery (25 °C)	%	98	≥75	T0662-2000
Residues after TFOT				
Mass change	%	−0.13	±1.0	T0609-2011
Penetration ratio (25 °C)	%	76.6	≥65	T0604-2011
Ductility (5 °C, 5 cm/min)	cm	31	≥15	T0605-2011

**Table 6:** Samples information.

Tests	Specimens	Replication
Penetration test	SBS00, SBS50, SBS100, HEA50, HEA100	4
Softening point test	SBS00, SBS50, SBS100, HEA50, HEA100	4
Ductility test	SBS00, SBS50, SBS100, HEA50, HEA100	4
Brookfield viscosity test	SBS00, SBS50, SBS100, HEA50, HEA100	3
Temperature sweep test	SBS00, SBS50, SBS100, HEA50, HEA100	3
Multiple stress creep and recovery test	SBS00, SBS50, SBS100, HEA50, HEA100	3
Bending beam rheological test	SBS00, SBS50, SBS100, HEA50, HEA100	3

## Data Availability

All data used during the study appear in the published article.
